# Excessive Crossed Disparity Detection by Visual Evoked Potentials to Reduce Visual Discomfort in 3D Viewing

**DOI:** 10.1155/2018/7098389

**Published:** 2018-11-01

**Authors:** Xiao Wang, Liuye Yao, Zhiyu Qian, Lidong Xing, Weitao Li, Yamin Yang

**Affiliations:** Department of Biomedical Engineering, Nanjing University of Aeronautics and Astronautics, Nanjing 210016, China

## Abstract

As excessive crossed disparity is known to cause visual discomfort, this study aims to establish a classification model to discriminate excessive crossed disparity in stereoscopic viewing in combination with subjective assessment of visual discomfort. A stereo-visual evoked potentials (VEPs) experimental system was built up to obtain the VEPs evoked by stereoscopic stimulus with different disparities. Ten volunteers participated in this experiment, and forty VEP datasets in total were extracted when the viewers were under comfortable viewing conditions. Six features of VEPs from three electrodes at the occipital lobe were chosen, and the classification was established using the Fisher's linear discriminant (FLD). Based on FLD results, the correct rate for determining the excessive crossed disparity was 70%, and it reached 80% for other stimuli. The study demonstrated cost-effective discriminant classification modelling to distinguish the stimulus with excessive crossed disparity which inclines to cause visual discomfort.

## 1. Introduction

**Figure 1:**
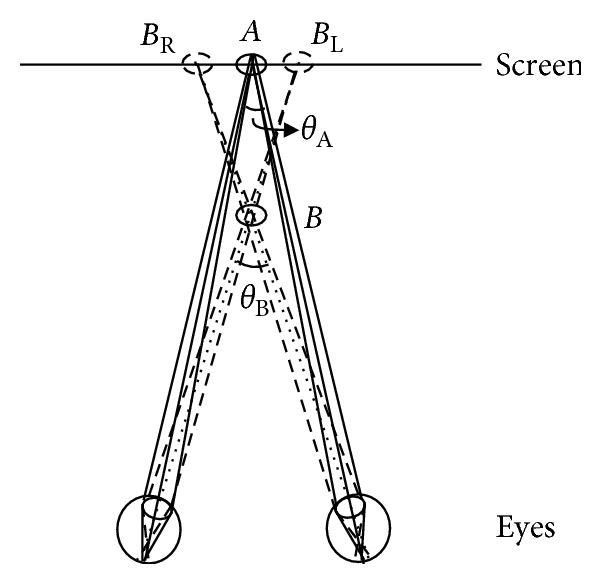
The vergence-accommodation conflict (VAC) and the disparity.

Stereoscopic images or videos may potentially create immersive experiences compelling viewers to believe they are physically present in a virtual environment. However, negative effects, including visual discomfort and visual fatigue, associated with immersive stereoscopic display have been reported [1–3]. Illustrated by Shibata et al. in 2011, vergence-accommodation conflict (VAC) is one of the key reasons that could cause visual discomfort. [4]. The vergence refers to the simultaneous eye movements in opposite directions that human could obtain the single binocular vision, and the accommodation is the change that optical power enters human eyes to maintain the sharpness of the image.  Figure 1 shows the basic principles of VAC. When people converge on point A on the screen, the vergence distance equals to the focal distance. However, when the eyes converge at point B that locates in front of the screen, the focal distance is still the same as that to point A while the vergence distance is shorter than the focal distance. In that condition, VAC is considering to increase. The monocular eye sees the point on the screen separately at *B*_L_ and *B*_R_. The distance between *B*_L_ and *B*_R_ is termed as horizontal parallax. The difference between the convergence angle at point A and point B, *θ*_A_−*θ*_B_, is defined as disparity (Figure 1). Clearly, the disparity at point A is zero (i.e., 2D image), and the disparity at point B is a negative value (i.e., the crossed disparity). The positive sign before the disparity magnitude refers to the location of the vergence that is at the back of the display screen (i.e., the uncrossed disparity). The magnitude of VAC depended on the image contents that were relative to the viewer's distance from the display [4]. If the magnitude of the disparity is too large, the crystalline lens in human eyes would strive to accommodate the difference between the focus and the vergence, so that the visual stress would increase, accompanying with visual discomfort.

Indeed, according to previous research, people were sensitive to crossed disparity which is easy to lead uncomfortable feelings [5, 6]. Suh et al. found that 3D images with crossed disparity caused greater degree of nearwork-induced transient myopia than 2D images did and had more significant effects on the development and progression of permanent myopia [7]. Chen et al. found decreased visual comfort was caused by crossed disparities in autostereoscopic display as viewing time increased [8]. Lambooij recommended that the value of disparity should adhere to a limit of 1° to guarantee visual comfort in consumer applications, such as stereoscopic television [9]. Jung et al. compared the brain activation of viewing uncomfortable videos with excessive screen disparities to that of comfortable videos with small screen disparities by functional magnetic resonance imaging (fMRI) and identified that the uncomfortable videos with excessive screen disparities evoked higher level activation in the right middle frontal gyrus (MFG), the right inferior frontal gyrus (IFG), the right inferior parietal lobule (IPL), the right middle temporal gyrus (MTG), etc. [10]. They concluded that visual discomfort due to excessive screen disparities was caused by sensory and/or motor phenomena that involved the intraparietal sulcus (IPS) regions, the frontal eye field (FEF), and premotor cortex [10]. Similarly, Kim et al. showed that high-fatigue caused by excessive binocular disparity intensified the IPS regions than the low-fatigue group did [11]. Visual evoked potential (VEP) measures the functional integrity of the visual pathways from retina via the optic nerves to the visual cortex [12] and could be obtained by the placement of electrodes at occipital lobe [12]. As it is known that VEP correlates closely to visual function, many studies have been made with the attempt to study stereoscopic vision by the use of VEP. Cheng et al. demonstrated the correlation between P1 component in VEP with the image brightness and proposed that whether the uncrossed disparity existed in a stimulus could be determined based on the latencies of N2, P3 components [13]. Wijeakumar et al. considered the change of N1 and P2 components as a complex component, and the enhanced N1-reduced P2 complex could be an indicator of binocular disparity in V1 [14]. In order to further elucidate the relevance between visual discomfort and VEP in stereo viewing, Negishi et al. compared the P100 component evoked by checkerboard pattern reversal stimulation before and after visual tasks and found that the latencies both delayed after the tasks in 3D presented and in real space. Although their result indicated that the latency of P100 could reflect the visual fatigue by vergence eye movement, it was not a 3D-specific factor [15]. Mun et al. indicated that 3D visual fatigue not only delayed P600 latencies but also significantly reduced P600 amplitudes thorough their steady-state visually evoked potential (SSVEP) experiment [16]. They also found that P4 and O2 electrodes showed significant fatigue effects in attended task with 8.57 Hz [16]. However, stimuli with disparity was not included in their study, and the SSVEP process required one-hour 3D viewing for conducting experiment. Previous studies have successfully proved the potentials of VEP as an effective indicator of disparity and as a detectable measure for assessing visual discomfort in 3D viewing, respectively. However, the effects of disparity and to which degree the disparity would evoke visual discomfort have not yet been well studied based on VEP.

Therefore, this paper developed a VEP experiment system and established a discriminant function based on visual comfort-related VEP results to distinguish the stimulus with excessive crossed disparity which inclines to cause visual discomfort. The classification model established in this study could potentially be useful for increasing fundamental knowledge towards the reduction and the precaution of the visual discomfort caused by disparity.

## 2. Materials and Methods

### 2.1. Stereo-VEP Experiment

**Figure 2:**
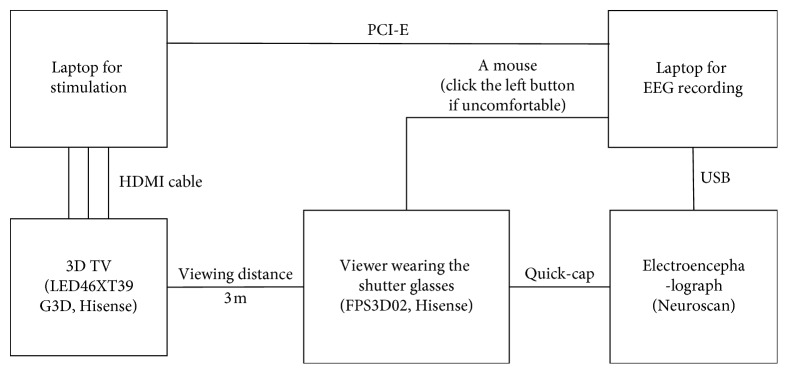
The block diagram of the stereo-VEP experimental system.

The block diagram of the stereo-VEP experimental system is shown in Figure 2. The 3D TV (LED46XT39G3D, Hisense) provided the visual stimuli to the viewer. The viewer watched the stimulations through a pair of 3D shutter glasses (FPS3D02, Hisense). The stimulation was generated in the laptop with E-prime 2.0 and was synchronously displayed on the 3D TV through an HDMI cable. The viewer faced to the centre of the 3D TV screen at a distance of 3 meters. The 32-lead Neuroscan EEG recorder was used to record the viewer's EEGs during the experiment. The distribution of the electrodes on the Quick-Cap was setup according to the expanded international 10–20 Montage system. The reference electrode was placed at the right mastoid M2. The sampling rate was 1 kHz, and the impedance of each electrode was lower than 5 kΩ. The laptop connected with the EEG recorder through the USB port. It monitored the EEG signals in real time and recorded the mark of the stimulus synchronously via the PCI Express (PCI-E) bus interface. A mouse was set for the viewer to report feedbacks of uncomfortable feelings.

**Figure 3:**
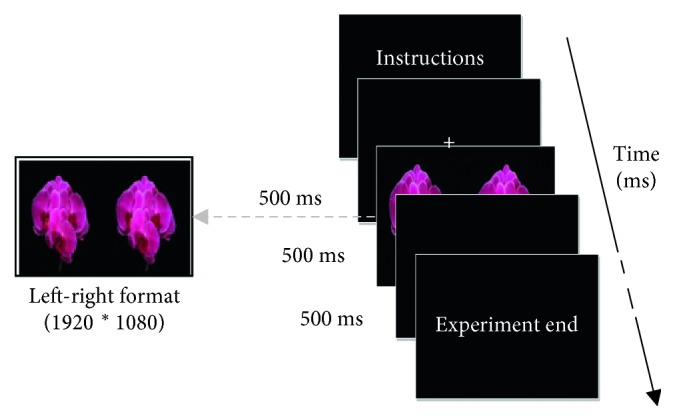
The stereo-VEP paradigm.

The “on-off” stereo-VEP paradigm is shown in Figure 3. In the paradigm, four images with different disparities were used as the stimuli.  Table 1 listed their disparity information. Disparity 0° means there is no horizontal position shift between the left and right sides in the image, the “+” sign means the disparity is an uncrossed disparity, and the “−” sign refers to crossed disparity. The resolution of the image was 1920 ∗ 1080. All stimuli were provided by Professor Qiu and his research group from the School of Arts in Peking University. When the experiment began, an experimental instruction appeared on the screen. When the viewer fully understood the instruction, he or she would press the space key to initiate following parts of the experiment. A cross was shown at the centre of the screen for five seconds to draw the viewer's attention. One of four images with different disparities would randomly display and remained for 500 ms for each, followed by a black background for 500 ms. If the viewer felt uncomfortable when they saw the current stimulus, he or she could click the left button on the mouse to report the uncomfortable feelings. One session of the experiment totally presented 240 trials (60 trials for every stimulus). All viewers participated in the experiment took two sessions continuously.

The experiment was conducted in a quiet room, and the temperature was kept at 24°C. Ten right-handed volunteers (male: 9, female: 1, age: 23 ± 2 years old) with normal stereoscopic vision participated in this experiment. A process was set before starting the experiment to test whether the viewers could correctly perceive the stimuli. The four stimuli appeared in the experiment presented to viewers one by one on the 3D TV. The viewers saw the stimuli through the active shutter glasses and then were required to orally report the general location of each stimulus. If the location they told was in accordance with the actual feature of the corresponding stimulus, then the viewer was regarded as the person without stereo blindness and allowed to participate in the following experiment. All volunteers were informed to sign a consent form before the experiment. All experiments were carried out in accordance with institutional guidelines of Nanjing University of Aeronautics and Astronautics (NUAA). All experimental protocols were approved by the Ethics Committee of NUAA.

### 2.2. Data Processing

The reference was changed to Cz electrode during offline processing. The baseline was corrected and the EEG data were filtered by a 50 Hz notch and a 0.01–30 Hz bandpass filter. The filtered EEG data were corrected by subtracting the eye movement artifacts using the covariance method. VEPs were obtained by averaging the time-locked and phase-locked EEGs without uncomfortable feedbacks. Valid VEPs data evoked by per stimulation were averaged over 90 trials.

### 2.3. Classification

After obtaining the VEP data of each viewer, the latency and the amplitude of various VEP components were selected by detecting the peak or valley in a certain short-time duration. Considering the statistic results and the VEP wave, six features of VEP were chosen to establish the classification model. The Fisher's linear discriminant (FLD) was used for this binary classification. Due to limitation of the sample size, the leave-one-out cross-validation (LOOCV) was used to estimate the classification error.

## 3. Results and Discussion

### 3.1. Results

**Figure 4:**
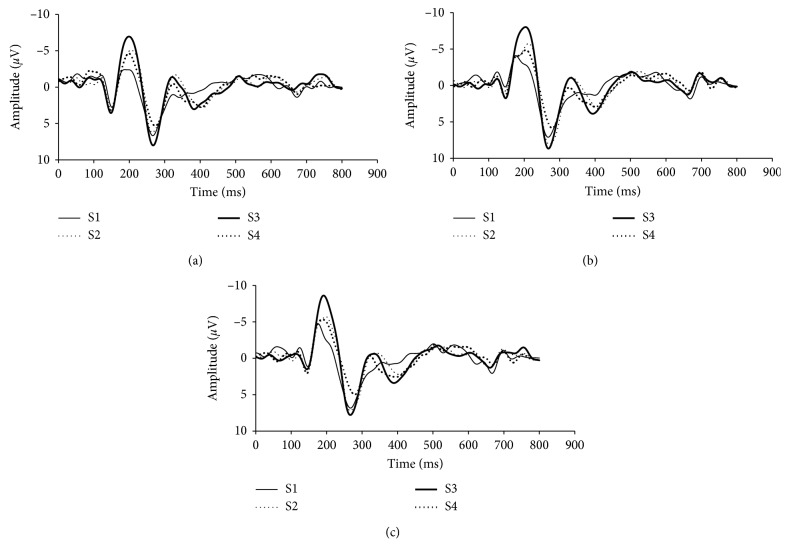
VEPs of 4 stimuli at O1, O2, and Oz electrodes from one person. (a) O1 electrode. (b) O2 electrode. (c) Oz electrode.

According to previous results [12], three electrodes (O1, Oz, and O2) at the occipital lobe were analyzed in present study. Typical VEPs evoked by four stimuli at O1, O2, and Oz electrodes from one individual are presented in Figures 4(a)–4(c). It is clear that the P3 components in all three electrodes were the most obvious and so were the C1 and C2 components in the O2 electrode. The Pearson correlation coefficient showed that the amplitude of P3 component had a midrelevance with the disparity (O1: Pearson correlation coefficient = −0.474, *P*=0.006 < 0.01; Oz: Pearson correlation coefficient = −0.480, *P*=0.005 < 0.01; O2: Pearson correlation coefficient = −0.459, *P*=0.008 < 0.01) and the paired *T*-test confirmed that the P3 component had significant difference between any two different types of visual stimuli (*P* < 0.05).

Six features for VEPs (O2 electrode: the latencies of P3 component and C2 component, the amplitude of C1 component; O1 electrode: the latency and amplitude of P3 component; Oz electrode: the amplitude of P3 component, termed as *x*_1_ to *x*_6_, respectively) were chosen for establishing the classification model to distinguish visual discomfort-related excessive crossed disparity. S3 was defined as class 1 which contains excessive crossed disparity and S1, S2, and S4 were defined as class 2.

Based on FLD results, the correct rate of the class 1 was 70%, and it reached 80% for class 2. 77.5% of cross-validated grouped cases were correctly classified (Wilks' lambda = 0.605, χ^2^=16.576, *P*=0.011 < 0.05).  Table 2 shows the count of correct and incorrect classification for each class. Equations (1) and (2) were the discriminant functions of class 1 and class 2.

The discriminant function of class 1:

(1) $$\begin{array}{c} y_{1}=-131.993-0.044x_{1}+0.158x_{2}+0.735x_{3}+0.787x_{4}+7.383x_{5}-2.372x_{6}. \end{array}$$

The discriminant function of class 2:

(2) $$\begin{array}{c} y_{2}=-126.581+0.153x_{1}+0.221x_{2}+0.346x_{3}+0.563x_{4}+7.975x_{5}-3.841x_{6}. \end{array}$$

As insufficient overlaps would generate small fluctuations to the amplitude and latency of components while acquiring ERP components, VEPs used for classification were overlapped by 90 trials for each person in present study. According to our previous experiments, we selected EEG data randomly and extracted 40 trials for every stimulus, from which stable EEG waves can be obtained. Afterwards, the new characters of VEP components were obtained and used for successful classification of stimulus with various disparity features within 0.000014 second (Table 3).

We also attempted multiclassification into four classes representing corresponding stimulus using VEP data by FLD. Stimuli S1 to S4 were named as class 1 to class 4, and nineteen features from VEP were used in this classification (O1 electrode: latency and amplitude of C1, C2, N2, and P3 components; Oz electrode: latency of C1, C2, and N2 components, amplitude of C1, C2, and P3 components; O2 electrode: latencies of N2 and P3 components, amplitude of C1, C2, and P3 components).  Table 4 listed the result of the multiclassification. The correct rates of class 1 to class 4 were, respectively, 60%, 80%, 60%, and 90%. Cross-validated grouped cases were classified with a correct rate of 72.5%.

**Figure 5:**
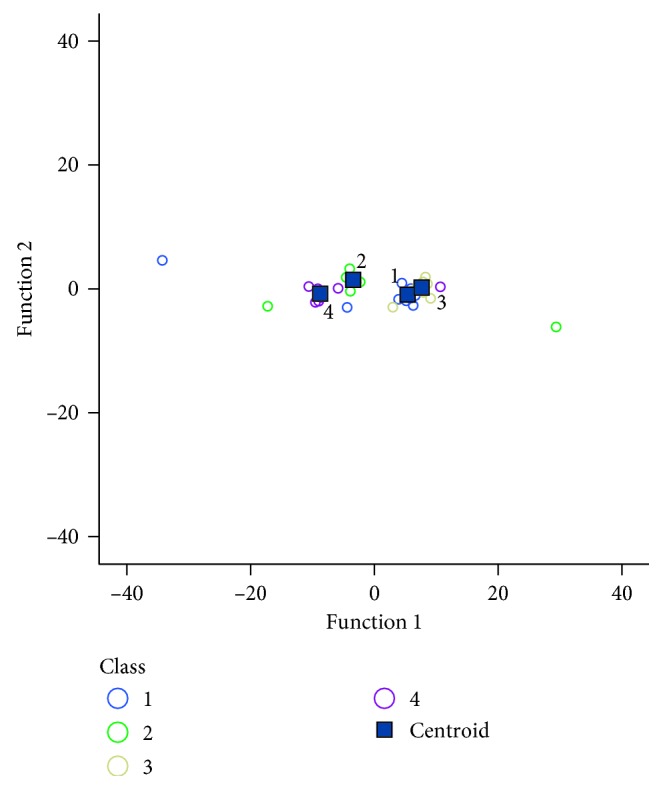
The centroids of four classes.

Figure 5 shows the centroids of four classes. The horizontal and vertical coordinates represent two discriminant functions that aim to projecting the features and classifying the projections. The figure clearly showed that the centroids of S1 and S3 located very close to each other, indicating that it was difficult to discriminate S1 from S3. As the sample size matters in multiclassification, more samples are required for each class to achieve better classification performance. It might be due to the less samples of each class in the multiclassification, the FLD performed better in the binary classification than in the multiclassification in this study.

### 3.2. Discussion

Due to the limitations of current binocular display technology, inappropriate disparities, such as the excessive crossed disparity, will cause visual discomfort when the viewers are perceiving the stereoscopic impression in the stereoscope system, including anaglyph 3D, polarized 3D, and active shutter system. Not only the traditional stereoscope system but also autostereoscopy (glasses-free 3D) display by exceptional 3D using autostereoscopic lenticular lens and parallax barrier is also closely related to disparity. Many existing 3D visual discomfort prediction models are based on the features extracted from computed disparity maps. For example, Sohn et al. proposed object-dependent disparity features to predict the visual discomfort in stereoscopic 3D images [17]. So et al. combined the strength and size of the excessive disparity range, the complexity of the background objects, the variation of the motion-depth, and the contrast of the objects in the scene to evaluate visual fatigue [18]. Ying et al. proposed a visual comfort assessment based on scene mode classification and showed that the proposed method performs higher assessment accuracy than some state-of-the-art methods [19]. Zellinger and Moser improved a visual discomfort predicting model by evolving the Haralick disparity contrast into the standard second-order statistical approach-based co-occurrence matrices, which performed better than before [20]. However, above methods of implementing the 3D visual discomfort model are relying on computed disparity maps and largely depend on the accuracy of the disparity result. Chen et al. provided a visual discomfort predicting model called percentage of unlinked pixels (PUP) which can be used to predict experienced 3D visual discomfort without explicit disparity calculation. Their results indicated that the predictive power attained by calculation of PUP maps was highly competitive with traditional disparity computation but with a higher calculation speed [21]. Other than traditional stereoscope system, autostereoscopic systems like integral imaging and holography are seemed to overcome the VAC problem [22]. However, the restruction quality becomes another factor relating to visual discomfort in these methods. Li et al. proposed the computational integral imaging (CII) method by the iterative perfect reconstruction technique to improve the visual quality of reconstructed 3D scenes, and their results showed that their method outperformed the conventional super-resolution reconstruction-based CII methods [23]. Nevertheless, relative complex algorithm and complicated pre-reconstruction process are usually required for the acquisition of stereoscopic 3D content with visual quality. It is thus necessary to build a 3D discomfort prediction model without explicit disparity calculation and with a relatively simple and easy-operating method. A cost-effective experimental system based on VEP while 3D images viewing was developed in present study for the assessment and classification of disparity-related visual discomfort. In the research of visual discomfort assessment, many researchers discriminated comfortable conditions from uncomfortable ones during stereoscopic vision by the analysis of EEG signals [24–26]. Frey et al. proved the feasibility of EEG for estimating visual comfort as the viewers watched stereoscopic displays. However, their subjective symptom questionnaires were recorded after each experimental session rather than immediately after the exposure of individual stimulus [27].

Previous studies have proved that VEP could be an effective indicator of the change of EEG signals induced by disparity or caused by visual discomfort [13–16]. According to Creel's report [12], VEP measures the functional integrity of the visual pathways from retina via the optic nerves to the visual cortex and could be obtained by the electrodes at occipital lobe. Jung's study [10] investigated the brain activities in different locations while viewing stereoscopic images with different screen disparities. They found that the crossed disparity near −1 degree activated the right inferior parietal lobule (IPL; BA 40) and the right middle frontal gyrus (MFG; BA 6), which was in accordance with Tsao's study [28] in 2003. Except the middle frontal gyrus, V3A belonged to the occipital lobe and IPL (BA40) located near the occipital lobe. Therefore, electrodes at the occipital lobe were chosen and expected to receive the EEG signals from the related regions for feature extraction of VEPs.

In our present study, the subjective discomfort feedback was captured after every stimulation without interrupting the experiment. Disparity beyond one degree is known to cause noticeable visual discomfort, and in consideration of the previous suggestion [9], stimulus with crossed disparity of −0.9 (S3) was used in present study. Although this range is within that typically considered as a comfortable depth budget in stereoscopic displays, based on our results, subjective discomfort feedbacks could still be recorded easily. The subjective feedback showed that S3 received the most discomfort feedbacks, which was far beyond the other stimuli. However, the total amount of discomfort feedbacks merely accounts for a very small portion of the cumulative total of the times of stimulations. Furthermore, there was no discomfort feedbacks recorded at the very beginning of each session. During offline data processing, VEPs evoked by different disparities were only extracted when the viewers were under comfortable state, so that the classification contributes to visual discomfort prevention. Only 6 features of VEP from 3 electrodes were used in the binary classification modelling. The features were easy to detect since VEP is well extracted. Comparing our model with previous studies, each experimental session in present study took less than 5 minutes, and the classification process did not include any complex algorithm.

VEP is a commonly used clinical visual diagnosis method, and its high temporal resolution owns potentials for expanding current strategy into a real-time condition in the future. Many researchers have devoted themselves into exploring the effective way of extracting the single-trial ERP and have made some achievements [29–31]. With further research on single-trial ERP extraction, the model in this study would be further optimized towards a real-time determination of excessive crossed disparity in stereoscopic content.

Generally, a VEP-based experimental system was developed to acquire both VEPs and subjective feedbacks while viewing 3D images with various disparity. The relevance between visual discomfort and disparity was determined by analyzing VEP results and a classification model was established for distinguishing stimulus with excessive crossed disparity which inclines to cause visual discomfort. Compared with previous studies, the subjective discomfort feedback was captured after every stimulation without interrupting the experiment, and VEPs evoked by different disparities were only extracted when the viewers were under comfortable state. The classification modelling by FLD without explicit calculation is more cost-effective for the assessment and classification of disparity-related visual discomfort.

## 4. Conclusions

The study established a classification model based on VEP and FLD to discriminate the excessive crossed disparity in stereoscopic images. Six features from three electrodes located at the occipital lobe were used in the binary classification modelling. The correct rates of classification to the excessive crossed disparity and the other class were separately 70% and 80%. The accuracy of the classifier reached 77.5%. The multiclassification was also attempted in this study; however, more samples are required for each class to achieve better classification performance. The classification model established in this study could potentially be useful for increasing fundamental knowledge towards the reduction and the precaution of the visual discomfort caused by disparity.

## Figures and Tables

**Table 1:** The disparity information of the stimuli.

Stimulus	Disparity (°)
S1	0
S2	±0.5
S3	−0.9
S4	+0.9

**Table 2:** The count of correct and incorrect classification for each class.

Class		Prediction		Total samples
		1	2	
Samples	1	7	3	10
(True class)	2	6	24	30

**Table 3:** The features overlapped by 40 trials and the result of classification.

Stimulus	Features						Class	
	*x*_1_ (ms)	*x*_2_ (ms)	*x*_3_ (*μ*V)	*x*_4_ (ms)	*x*_5_ (*μ*V)	*x*_6_ (*μ*V)	True class	Prediction
S1	259	126	−1.971	259	6.165	6.924	2	2
S2	275	125	−3.254	281	7.263	6.672	2	2
S3	280	126	−2.557	287	12.6	12.02	1	1
S4	289	120	−3.45	286	8.147	8.077	2	2

**Table 4:** The result of 4-class classification.

Class		Prediction				Total samples
		1	2	3	4	
Samples (true class)	1	6	0	4	0	10
	2	0	8	0	2	10
	3	4	0	6	0	10
	4	0	1	0	9	10

## Data Availability

The data used to support the findings of this study are available from the corresponding author upon request.
